# Special Issue: New Insight into the Molecular Role of Lipids and Lipoproteins in Vascular Diseases

**DOI:** 10.3390/ijms241310659

**Published:** 2023-06-26

**Authors:** Sonia Benitez, José Luis Sánchez-Quesada

**Affiliations:** 1 Cardiovascular Biochemistry Group, Institut d’Investigació Biomèdica Sant Pau (IIB SANT PAU), 08041 Barcelona, Spain; 2 CIBER of Diabetes and Related Metabolic Diseases (CIBERDEM), Instituto de Salud Carlos III, 28029 Madrid, Spain

Lipids and lipoproteins play a key role in cardiovascular diseases (CVD), mainly in the development of atherosclerosis. It is widely accepted that an elevated concentration of low-density lipoproteins (LDL) in circulation is a risk factor for CVD, whereas high concentrations of high-density lipoproteins (HDL) have been related to cardioprotection. However, despite the achievement of proper lipid and lipoprotein concentrations by medical treatments, CVD remain major causes of death and disability. This suggests that not only is the quantity of lipids important, but so is their quality and distribution among lipoproteins. In this context, beyond the lipid profile, studies attempted to ascertain the composition and functionality of lipoproteins and their relationship with other parameters related to atherosclerosis, and vascular risk are also essential. In this manner, studying the value of lipoprotein-related molecules as biomarkers and elucidating the ensuing activated molecular mechanisms could promote the discovery of novel targets and new therapies for CVD.

This Special Issue “New Insight Into the Molecular Role of Lipids and Lipoproteins in Vascular Diseases” of the International Journal of Molecular Sciences aims to update novel knowledge regarding the qualitative properties of lipids and lipoproteins in the context of vascular diseases from different perspectives. The issue includes two reviews that update and provide new information about two modified forms of LDL: oxidized LDL (oxLDL) and electronegative LDL (LDL(−)). This Special Issue also contains five other original articles. One article, which describes a new enzymatic activity associated with LDL(−), is closely related to the review focused on this particle. In two original articles, the alterations in the qualitative properties of lipoproteins in two pathologies with high CVD incidence, such as HIV and metabolic syndrome (MS), are described. The evaluation of such alterations could be useful in predicting vascular risk. In this line, another article evaluates the value of lipid atherogenic indices as predictors of vascular calcification, which is a determinant of cardiovascular risk. Finally, another study reports a novel anti-inflammatory property of PCSK9 inhibitors, in parallel, with changes in lipid parameters. The common point of all these studies is to provide a novel approach to cardiovascular risk, either by analyzing the qualitative characteristics of lipoproteins, evaluating the use of ratios between the different lipoprotein fractions, or using nuclear magnetic resonance (NMR) to obtain more detailed information about the lipoprotein profile.

It is well known that the modification of LDL by different mechanisms, including oxidation, is related to the initiation and progression of atherosclerosis, since modified LDLs are crucial inductors of lipid accumulation and inflammation in the artery wall. Oxidized LDLs (oxLDLs) have been detected in atherosclerotic lesions. The review by Itabe et al. [1] discusses the knowledge about oxidized lipoproteins from a novel standpoint to elucidate the origin and properties of oxLDL in vivo. Due to the difficulty of isolating oxLDL from human plasma, the features of in vivo oxLDL, including the chemical analysis of the oxidation products and their biological and metabolic properties, were proposed by analyzing in vitro modified lipoproteins and LDL subfractions. In this regard, copper-induced oxLDL has been widely used as the standard model of oxLDL. In vivo forms of lipoproteins, such as small dense LDL, Lp(a), and LDL(−), also mimic several oxLDL-induced deleterious effects and are comprehensively revised.

In the 1990s, the availability of enzyme-linked immunosorbent assay (ELISA) procedures developed with monoclonal antibodies (mAbs) that recognize several epitopes of oxLDL allowed the measurement of oxLDL levels in biological samples. This discovery opened a new line of clinical research focused on the relationship between oxLDL levels and pathologies associated with CVD. Despite all this evidence, the use of plasma oxLDL levels as a biomarker for CVD and its actual role in vivo is still a controversial issue [2]. Interestingly, this review also discusses the possible role of oxidized HDL (oxHDL) and oxLDL–oxHDL complexes in the development of atherosclerosis. Further studies on oxidized in vivo lipoproteins, approaching the molecular analysis of modified structures, could elucidate their actual features.

LDL(−) is a minor modified LDL subfraction present in circulation with atherogenic characteristics [3]. Accordingly, its proportion is increased in acute CVD and in pathologies associated with vascular risk, such as dyslipidemia or diabetes. LDL(−) has been proposed as a biomarker for CVD and, thus, it is proposed as a putative target to prevent clinical events. However, the heterogeneous nature of LDL(−) hampers the knowledge of the mechanisms related to its origin and its deleterious effects. In this Special Issue, Puig et al. address the origin of enzymatic activity involved in the previously reported increased sphingosine (Sph) content in LDL(−) [4]. Using different experimental approaches, the authors reveal that their own LDL(−) particle, but not native LDL, shows ceramidase (CDase)-like activity. This enzymatic activity led to ceramide hydrolysis, with an ensuing increase in the content of Sph and free fatty acids in LDL(−). The existence of this hitherto unknown enzymatic activity in LDL(−) is of great interest, since no CDase activity had been found previously in any lipoprotein. Unfortunately, the presence of a specific protein with known CDase activity was not detected. In this regard, future studies are warranted to ascertain the origin of this activity, whose targeting could be useful in preventing the harmful effects of LDL(−) on cells.

In close relationship with the findings of Puig et al., the involvement of this CDase-like activity as a part of a multienzymatic complex in LDL(−), together with other phospholipolytic activities, is hypothesized by Benitez et al. [5]. Besides the CDase activity described in this Special Issue, LDL(−) was reported to show platelet activating factor acetyl hydrolase (PAF-AH) and phospholipase C (PLC)-like activities, whereas these enzymes are absent or very scarce in native LDL. Based on the complementarity of the products and substrates of these different enzymatic activities, the authors speculate that these activities could act in a concerted manner in LDL(−). The authors hypothesize that PLC-like and CDase-like activities could be generated by conformational changes in apoB-100 that allow the interaction among them in proximity to the PAF–AH active site. Although the evidence suggests that the coordinated action of these enzymes represents a counteracting mechanism to avoid LDL(−)-induced deleterious biological effects, the final effect is still unknown and deserves further investigation.

Metabolic syndrome (MS) is a risk factor for CVD. These patients not only have an altered lipoprotein profile, but they also have modifications in the qualitative properties of lipoproteins, including loss of function in HDL [6]. HDL contributes to maintaining proper endothelial function through its anti-oxidative and anti-inflammatory effects and promoting NO production. MS patients show endothelial dysfunction with impaired NO production and increased endothelial lipase (EL) levels. EL is an enzyme produced by the endothelium, with seemingly protective effects, such as the regulation of HDL cholesterol levels, and the improvement of the qualitative properties of HDL. Klobucar et al. evaluated the association between HDL properties, endothelial function, and serum EL levels in controls and MS patients [7]. The study found that the presence of MS modulates the association between HDL and endothelial function, as well as between EL and HDL. The authors proposed that EL-mediated depletion of HDL phospholipids might be a driving factor for the negative association of phospholipids in HDL with endothelial function in controls. More studies are needed to ascertain the potential clinical use of blocking EL as a therapeutic target in patients with MS or other pathologies associated with impaired HDL function.

In the study by Rehues et al., a different approach is taken, with the focus on the inflammatory phenomena associated with arteriosclerosis [8]. The authors analyzed the effect of the treatment with PCSK9 inhibitors in patients with familial hypercholesterolemia. In this study, the authors used NMR to measure lipoprotein and lipid levels, as well as novel inflammation markers. As expected, PCSK9 inhibitors change the plasma levels of total cholesterol and LDL cholesterol, but they also have an important effect in reducing the concentration of the new inflammation markers: glycA, glycB, and glycF. These markers are glycoprotein peaks, detectable by NMR, whose concentration in plasma is altered under inflammatory conditions [9]. The authors demonstrated that the beneficial effect on these inflammation markers is not direct, but it is associated with a decrease in the plasmatic concentration of triglyceride and apoC-III. Thus, the authors point to apoC-III as a very important modulator of systemic inflammation that often accompanies patients with underlying arteriosclerosis. Thus, this study reaffirms the close connection between alterations in lipoprotein components and the appearance of inflammatory phenomena.

Masip et al. also use the powerful 1H-NMR tool to obtain advanced characterization of the lipid profile, including the size and number of HDL, LDL, and VLDL particles [10]. In this case, the study was conducted on patients with HIV undergoing antiretroviral therapy (ART). From an immunological point of view, some of these patients have an appropriate response to therapy, but other patients do not respond to this therapy and present increased cardiovascular risk, in part due to impaired immune function. The authors found that ART treatment dissipates the basal differences between the lipoprotein profiles of immunological non-responders and immunological responders. An important increase in LDL and HDL particles at month 36 was observed in both groups of HIV patients.

Finally, the study by Dai and colleagues uses a different strategy [11]. Instead of using complex methods (such as NMR) or analyzing certain qualitative changes in lipoproteins (modified lipoproteins, glycoprotein markers), they evaluated a series of different lipoprotein-based atherogenic indices as putative predictors of artery calcification, previously measured by fluorine 18–sodium fluoride (^18^F-NaF) PET/CT. This approach is interesting because it is based on methods to determine the lipid profile that are simple, cheap, and accessible to most health centers. Thus, they analyzed the following ratios: Non-HDL Cholesterol (TC-HDL cholesterol), Castelli’s Risk Index I (CRI-I, TC/HDL cholesterol, Castelli’s Risk Index II (CRI-II, LDL/HDL cholesterol), Atherogenic Coefficient (AC, Non-HDL/HDL cholesterol), and Atherogenic Index of Plasma (AIP, log (TG/HDL cholesterol)). The authors concluded that these atherogenic indices are better predictors of vascular calcification than standalone lipid metrics. Hence, atherogenic indices would provide clinicians with a more holistic understanding of atherosclerosis development over standalone lipoprotein profile metrics [12].

Although different therapies can control the plasma levels of lipids and lipoproteins, there is a residual vascular risk that persists even after regulating the lipid levels. The qualitative properties of lipoproteins have emerged as essential factors determining vascular risk. Some of these alterations can lead to modified molecules that can become biomarkers for CVD. Searching for reliable and easy-to-determine plasma biomarkers is of great interest. In this Special Issue, different approaches are used to define the role of putative lipid/lipoprotein-related biomarkers (modified LDLs, atherogenic indices, inflammation-related molecules). At the same time, knowledge of the molecular mechanisms activated by these molecules is crucial to design strategies to target them with a benefit for CVD. Based on the articles included in this Special Issue, the decrease in glycoprotein synthesis by PCSK9 inhibitors, the inhibition of LDL(−)’s effects or the blocking of EL to improve HDL’s function could be useful approaches for decreasing cardiovascular risk.
